# Combination of susceptibility gene and traditional risk factors might enhance the performance of coronary heart disease screening strategy

**DOI:** 10.18632/oncotarget.16692

**Published:** 2017-03-29

**Authors:** Shiyan Nian, Lei Feng, Yang Zhao, Feng Luo, Shu Zhang, Dan Li, Wenbo Xu, Xingfeng Zhang, Dan Ye, Xuejing Bai

**Affiliations:** ^1^ Intensive Care Unit, People’s Hospital of Yuxi City, Yuxi, Yunnan, P.R. China; ^2^ Department of Laboratory, People’s Hospital of Yuxi City, Yuxi, Yunnan, P.R. China; ^3^ Department of Laboratory, The Sixth Affiliated Hospital of Kunming Medical University, Yuxi, Yunnan, P.R. China

**Keywords:** coronary heart disease, serum biochemical indices, susceptibility gene, screening strategy, performance

## Abstract

Coronary heart disease (CHD) associated risk factors and susceptibility genes were studied in parallel for decades, however, the combination of the classic CHD risk factors and genetic risk factors has been rarely studied. Previously; we reported that a single nucleotide polymorphism (SNP) in the stromal cell-derived factor 1 (SDF-1) gene was associated with CHD risk; in addition, we also established a CHD screening strategy using traditional CHD risk factors as independent variables. To explore how to combine genetic factors and traditional risk factors in CHD screening strategy, the CHD probabilities were tested in 218 males and 121 females according to their stromal cell-derived factor 1 (SDF-1) genotypes using CHD screening equations we reported previously. The genotypes had not altered the distribution characteristics of age, high-density lipoprotein cholesterol (HDL-C), triglyceride (TG), lipoprotein(a) (LP(a)), homocysteine (HCY) and total bilirubin (TBil) in males and age, HDL-C, HCY and γ-glutamyl transpeptidase (GGT) in females among genotypes. However, the mean CHD probability of subjects with G/G genotype was significantly higher than that of subjects with A/A genotype (0.51 ± 0.35 vs. 0.31 ± 0.31, *P* = 0.035). The mean CHD probability of subjects with G homozygous and G heterozygote was 0.48 ± 0.34 which displayed a difference trend to that of subjects with A homozygous (0.31 ± 0.31, *P* = 0.059). Our data suggested that genetic risk factors might be used as a classification standard to improve current CHD screening strategies.

## INTRODUCTION

Coronary heart disease (CHD) remains a persistent public health burden worldwide [1, 2]. The Global Burden of Disease report showed that ischemic cardiomyopathy was the leading cause of death, accounting for as much 12.2% of all deaths worldwide, with 7.2 million deaths in 2004 [1, 2]. CHD has been the focus of global medical research for almost a century [3]. Epidemiological studies have identified that age, sex, family history and ethnicity are classic un-modifiable risk factors and hypertension, dyslipidemia and diabetes are classic modifiable risk factors [4–10].

Single nucleotide polymorphisms (SNPs) are also widely studied in individual’s genetic predisposition for CHD [11]. Genome-wide association studies (GWAS) have identified more than 100 gene variants that are associated with CHD as reported in the National Human Genome Research Institute catalog [12]. These studies advance our understanding on the pathogenesis of CHD, and greatly improved the prevention, diagnosis as well as treatment on CHD. Recently, our group had reported that a SNP (rs1801157) in the stromal cell-derived factor 1 (SDF-1) gene (located at q11.21 on chromosome 10) is associated with CHD in our Chinese [13]. Multivariable logistic regression analysis showed that the onset of CHD in population with G/G genotype is 2.31 times higher than that in population with A/A genotype [13].

Meanwhile, a number of serum biochemical markers, which reflect dynamic physiological and pathophysiological processes within the body, had been found to be associated with CHD [7–10]. Our previous study showed that integrated serum biochemical indices may be used to screen for suspected CHD in our Chinese [14–16]. The raw or logarithm transformed age, high-density lipoprotein cholesterol (HDL-C), triglyceride (TG), lipoprotein(a) (LP(a)), homocysteine (HCY) and total bilirubin (TBil) could be used to screen CHD risk in a equation with distinct coefficient in males [14]. The raw or logarithm transformed age, HDL-C, HCY and γ-glutamyl transpeptidase (GGT) could be used to screen CHD risk in another equation with distinct coefficient in females [14].

Although the predicting strategy has evolved remarkably and more and more CHD associated SNPs were reported, the combination of the classic CHD risk factors and genetic risk factors has been rarely studied. The issues regarding how to combine the classic risk factors with genetic risk factors and whether genetic risk factors could further improve the performance of the predicting strategies using classic CHD risk factor are worthy of attention. In this report, our screening equations for suspected CHD using integrated serum biochemical indices were tested in 218 males and 121 females according to their SDF-1 genotypes retrospectively.

## RESULTS

### Distribution characteristics of gender specific traditional CHD risk factors, CHD probability and SDF-1 genotypes in CHD patients and controls

Our previous study showed that CHD probability for males and females could be calculated using following equations [14, 16]:

Probability(CHDmale final)= 1/{1+exp-(−6.846+0.102age-3.316HDL-C-0.677lnTG+

0.481lnLp(a)+2.195lnHCY-0.725lnTBIL)};

Probability(CHDfemale final)= 1/{1+exp-(−15.356+0.180age-3.105HDL-C+2.938

lnHCY+0.904lnγ-GT)}.

Table 1 showed the distribution characteristics of above gender specific traditional CHD risk factors. Of males; age, HDL-C, Lp(a) and HCY were significantly higher in CHD patients than these in controls; conversely, TBil was significantly lower in CHD patients than that in controls; TG level displayed a difference trend between CHD patients and controls; the CHD probabilities of CHD patients and controls calculated using above variables were 0.87 ± 0.17 and 0.30 ± 0.24 respectively. Of females; age, HDL-C, HCY and GGT were significantly higher in CHD patients than these in controls; the CHD probabilities of CHD patients and controls calculated using above variables were 0.87 ± 0.19 and 0.21 ± 0.26 respectively.

**Table 1:** Distribution characteristics of gender specific CHD risk factors, CHD probability and SDF-1 genotypes in CHD patients and controls

	CHD	Control	*P*
*Male (n = 218); n for CHD, 66; n for Control, 152*			
Age (yr)	62.5 ± 10.4	35.0 ± 9.2	<0.001
HDL-C (mmol/L)	1.14 ± 0.26	1.03 ± 0.28	0.008
TG (mmol/L)	1.52 (1.19, 2.20)	1.81 (1.04, 3.05)	0.092
Lp(a) (mg/L)	194.00 (80.00, 338.50)	79.00 (37.50, 177.00)	<0.001
HCY (umol/L)	22.00 (14.25, 26.00)	14.00 (11.00, 17.00)	<0.001
TBil (umol/L)	10.10 (7.53, 12.05)	13.10 (10.10, 18.05)	<0.001
CHD Probability	0.87 ± 0.17	0.30 ± 0.24	<0.001
G/G, n (%)	41 (62.1)	74 (48.7)	0.046
G/A, n (%)	23 (34.8)	66 (43.4)	0.151
A/A, n (%)	2 (3.1)	12 (7.9)	0.147
*Female (n = 121); n for CHD, 20; n for Control, 101*			
Age (yr)	62.0 ± 9.1	32.2 ± 8.5	<0.001
HDL-C (mmol/L)	1.27 ± 0.27	1.05 ± 0.25	0.003
HCY (umol/L)	18.50 (13.50, 24.75)	14.00 (11.00, 16.00)	0.001
GGT (IU/L)	31.00 (19.25, 91.75)	21.00 (14.00, 34.50)	0.012
CHD Probability	0.87 ± 0.19	0.21 ± 0.26	
G/G, n (%)	9 (45.0)	46 (45.5)	0.581
G/A, n (%)	9 (45.0)	45 (44.6)	0.580
A/A, n (%)	2 (10.0)	10 (9.9)	0.627

Normally distributed data were presented as means ± standard deviation (SD), skewed data were presented as the median (interquartile range). Differences between groups were examined by using t test and Fisher exact probability test according to the data distribution tendency. Abbreviations: SDF-1, stromal cell-derived factor 1; TG, triglyceride; HDL-C, high density lipoprotein cholesterol; Lp(a), Lipoprotein(a); HCY, homocysteine; TBil, total bilirubin; GGT, gamma-glutamyl transpeptidase.

Of the SDF-1 genotypes; the percentage of G/G genotype in male CHD patients (62.1%) was significantly higher than that in male controls (48.7%); no other difference was found between CHD patients and controls (Table 1).

### Genotypes have not changed the distribution tendency of classic CHD risk factors in males and females

To learn whether SDF-1 genotype will alter the distribution tendency of CHD risk factors in males and females, the study subjects were pooled together and regrouped by their SDF-1 genotypes. As showed in Table 2, of males, no significant difference was observed between G/G, G/A and A/A genotypes regarding age, HDL-C as well as logarithm transformed TG, Lp(a), HCY and TBil. Similarly, as showed in Table 3, of females, no significant difference was observed between G/G, G/A and A/A genotypes regarding age, HDL-C as well as logarithm transformed HCY and GGT.

**Table 2:** Distribution tendency of the male-specific risk factors by genotypes

	G/G	G/A	A/A	*P*
	(*n* = 115)	(*n* = 89)	(*n* = 14)	
Age (yr)	44.6 ± 16.5	42.3 ± 14.2	35.4 ± 18.0	*0.100*
HDL-C (mmol/L)	1.05 ± 0.26	1.08 ± 0.30	1.06 ± 0.25	*0.794*
lnTG	0.57 ± 0.68	0.59 ± 0.64	0.51 ± 0.66	*0.922*
lnLp(a)	4.64 ± 0.97	4.61 ± 1.16	4.57 ± 0.98	*0.962*
lnHCY	2.75 ± 0.43	2.66 ± 0.44	2.73 ± 0.46	*0.319*
lnTBil	2.50 ± 0.42	2.47 ± 0.45	2.42 ± 0.52	*0.766*

Data were presented as means ± SD. Abbreviations: TG, triglyceride; HDL-C, high density lipoprotein cholesterol; Lp(a), Lipoprotein(a); HCY, homocysteine; TBil, total bilirubin; ln, logarithm transformed. Differences among groups were examined by using Kruskal-Wallis H test, one-way ANOVA, χ2 tests according to the data distribution tendency.

**Table 3:** Distribution tendency of the female-specific risk factors by genotypes

	G/G	G/A	A/A	*P*
	(*n* = 55)	(*n* = 54)	(*n* = 12)	
Age (yr)	37.7 ± 14.3	36.2 ± 13.6	38.8 ± 16.6	*0.797*
HDL-C (mmol/L)	1.12 ± 0.26	1.07 ± 0.29	0.99 ± 0.16	*0.254*
lnHCY	2.66 ± 0.36	2.59 ± 0.28	2.72 ± 0.33	*0.309*
lnGGT	3.24 ± 0.74	3.13 ± 0.65	3.04 ± 0.63	*0.548*

Data were presented as means ± SD. Abbreviations: HDL-C, high density lipoprotein cholesterol; HCY, homocysteine; GGT, gamma-glutamyl transpeptidase; ln, logarithm transformed.

### Male subjects with G allele displayed higher probability of CHD

Although above analysis showed that SDF-1 genotypes have not changed the distribution tendency of CHD risk factors in males and females, we are still not sure whether the genotypes will alter the CHD probability calculated by the following equation:

Probability(CHDmale final)= 1/{1+exp-(−6.846+0.102age-3.316HDL-C-0.677lnTG+ 0.481lnLp(a)+2.195lnHCY-0.725lnTBIL)}.

To clarify this issue, the pooled male subjects were regrouped by their SDF-1 genotypes, the mean CHD probabilities of each genotype group were calculated. As showed in Table 4; although the mean probabilities of each genotype group were low; the mean CHD probability of G/G genotype group was significantly higher than that of A/A genotype group (0.51 ± 0.35 vs. 0.31 ± 0.31, *P* = 0.035). The mean CHD probability of G homozygous and G heterozygote was 0.48 ± 0.34 which displayed a difference trend to that of A homozygous (0.31 ± 0.31, *P* = 0.059).

**Table 4:** Male subjects with G allele displayed higher probability of CHD

	*n*	Mean Probability	*P*
*G homozygous vs. A homozygous*			
G/G	115	0.51 ± 0.35	*0.035*
A/A	14	0.31 ± 0.31	
*G homozygous and G heterozygote vs. A homozygous*			
G/G + G/A	204	0.48 ± 0.34	*0.059*
A/A	14	0.31 ± 0.31	

### G allele did not increase the probability of CHD in female subjects

To learn whether G allele will alter the CHD probability in females, the female subjects were also regrouped by SDF-1 genotypes, the CHD probability calculated by the following equation:

Probability(CHDfemale final)= 1/{1+exp-(−15.356+0.180age-3.105HDL-C+2.938 lnHCY+0.904lnγ-GT)}.

As showed in Table 5, the mean CHD probabilities of G/G and A/A genotype groups were 0.33 ± 0.36 and 0.38 ± 0.43, respectively, no significant difference was observed between two genotype groups.

**Table 5:** G allele did not increase the probability of CHD in female subjects

	*n*	Mean Probability	*P*
*G homozygous vs. A homozygous*			
G/G	55	0.33 ± 0.36	*0.701*
A/A	12	0.38 ± 0.43	
*G homozygous and G heterozygote vs. A homozygous*			
G/G + G/A	109	0.31 ± 0.35	*0.524*
A/A	12	0.38 ± 0.43	

The mean CHD probability of G homozygous and G heterozygote was 0.31 ± 0.35 which displayed no significant difference to that of A homozygous (0.38 ± 0.43).

## DISCUSSION

Classic risk factors of CHD, such as age, sex, hypertension, dyslipidemia, and diabetes, have studied nearly for a century [3–10]. Now, we can try to predict CHD risk using several well-known prognostic models including equations derived from the Framingham Heart Study [17], the Women’s Health Study [18], the Prospective Cardiovascular Münster study [19] and the Systematic Coronary Risk Evaluation project [20]. Recently, the atherosclerotic cardiovascular disease risk in China project has also developed effective tools with good performance for ten-year atherosclerotic cardiovascular disease risk prediction among Chinese population [21]. Yet, these CHD risk estimation strategies still confined to traditional risk factors and its practical application is still need to be improved. The directions for improvement include inclusion of new risk factors and combination of genetic factors. This report has the following merits: firstly, our models including markers of liver function and HCY; secondly, we have tried the combination of SDF-1 genotypes with the biochemical risk factors. The ideal value of our equations to predict CHD is 1.00; the closer to 1.00 the higher the risk, the closer to 0.00 the lower the risk. In clinical practice, we do not know whether a visitor we met is a CHD patients or not, so we pooled the CHD patients and controls together to compare the mean CHD probabilities of each SDF-1 genotype. In males, the G/G genotype subjects are at significantly higher CHD probability than that of A/A genotype subjects. G homozygous and G heterozygote subjects also showed a higher tendency of CHD probability when compared with A homozygous subjects. The above phenomenon was not observed in female subjects, this might be explained by sex difference. In conclusion, our data provided an idea for the combination of genetic factors and traditional risk factors in the future.

SDF-1, i.e. C–X–C motif chemokine 12 (CXCL12), is a chemokine that encoded by the CXCL12 gene [22, 23]. SDF-1 is induced by proinflammatory stimuli such as tumour necrosis factor, lipopolysaccharide or interleukin-1 and may activate T-lymphocytes. SDF-1 activates C–X–C chemokine receptor type 4 to induce a rapid and transient rise in the level of chemotaxis and intracellular calcium ions [22–25]. SDF-1 may also bind to atypical chemokine receptor 3, which activates theβ-arrestin pathway and acts as a scavenger receptor for SDF-1 [22–25]. Thus, SDF-1 plays an important role in host inflammatory responses [22–25]. SNP at rs1801157 in the 3’-untranslated region [13]. A allele is regarded as a target of cis-acting factors, has been shown to up-regulate the expression of CXCL12 [26]. Our previous study found that G/G genotype of SDF-1 gene was associated with higher risk of CHD [13]. In this report, subjects with SDF-1 G/G genotype have elevated slightly in the CHD probability, this might also be explained by that the main role of SDF-1 is to modulate inflammatory responses but not metabolic physiology.

Although our models used to screen CHD patients were derived from large samples with rigorous statistical analysis, the main disadvantage of our study is the mismatching of age between cases and controls. In addition, our models were developed from a cross-sectional study, the results need further validation using a prospective study.

## MATERIALS AND METHODS

### Ethics issues

The Review Board of the Sixth Affiliated Hospital of Kunming Medical University (People’s Hospital of Yuxi City, Yuxi, China) approved the protocol of this study (approval number: YNYXH2010-0012; 1 May 2010). Written informed consent was obtained according to the guidance of the Chinese National Ethics Regulation Committee. Participants were simultaneously informed of their right to repeal consent by themselves or their kin, caretakers, or guardians.

### Subjects

As we reported previously, 339 local Chinese (218 males and 121 females) were recruited consecutively at the People’s Hospital of Yuxi City between September 2010 and December 2012 to assess the association between SDF-1 gene polymorphisms and presence of CHD. Of the 339 subjects, 86 and 253 were CHD patients and healthy controls, respectively. Patients were diagnosed with CHD according to American Heart Association guidelines [27]. All patients were confirmed by the obstruction of at least 1 large epicardial coronary artery by atheromatous plaque using coronary angiography [13]. Patients who met the exclusion criteria will be exclude from this study: alcohol abuse, diabetes, a history of smoking, chronic lung disease, xanthelasma, and evidence of noncoronary atherosclerotic disease. The 253 healthy controls did not have a history of chronic disease, autoimmune disease, or cardiovascular disease. A questionnaire was self-completed by all participants; the questionnaire includs ethnicity, sex, age, family history, medical history, smoking status and alcohol abuse.

### Polymorphism genotyping

As we reported previously [13], SNP at rs1801157 of SDF-1 gene was identified using the MassARRAY® system (Sequenom, San Diego, CA, USA). The MassARRAY® system is based on single base primer extension technology. MassARRAY® technology uses matrix-assisted laser desorption ionization time-of-flight mass spectrometry to measure the mass of the extension product(s) directly and to correlate the detected mass with a specific genotype. A 293-bp SDF-1 fragment (covering rs1801157) was PCR-amplified using the primers F (5’-CAGTCAACCTGGGCAAAGCC-3’) and R (5’-CCTGAGAGTCCTTTTGCGGG-3’) (GenBank accession number: L36033). The extension primer sequence was 5’-GCCCTCCCAGAAGAGGCAGACC-3’. For details on the protocol, please refer to “SNP Genotyping Using the Sequenom MassARRAY® iPLEX Platform (http://www.sequenom.com/)”.

### Clinical data collection

A fasting blood sample was collected from each participant in the morning of the day on which he or she visited our hospital. Serum lipid profiles, including triglycerides (TG), total cholesterol (TC), high-density lipoprotein-cholesterol (HDL-C), low-density lipoproteincholesterol (LDL-C), and apolipoprotein (apo)A, apoB, apoE, and lipoprotein (Lp) (a); indicators of kidney function, including UA; indicators of liver function, including gamma-glutamyl transpeptidase (GGT), total bilirubin (TBil), indirect bilirubin (IBil), and direct bilirubin (DBil); and HCY levels, were measured in our hospital’s laboratory using routine procedures.

Waking blood pressure was measured in the morning using a mercury sphygmomanometer. Hypertension was defined as a systolic pressure ≥140 mmHg or diastolic pressure ≥90 mmHg according to the American Society of Hypertension guidelines [28].

### Statistical analysis

All analyses were performed using SPSS for Windows software (ver. 18.0; SPSS Inc., Chicago, IL, USA) and the significance level (alpha) was set at 0.05. Differences between groups were explored using the Kruskal-Wallis H test or one-way ANOVA, depending on the distribution tendency of the data. According to our previous report and doctoral thesis, the CHD probability for male and female was calculated using following equations [14, 16]:

Probability(CHDmale final)= 1/{1+exp-(−6.846+0.102age-3.316HDL-C-0.677lnTG+0.481lnLp(a)+2.195lnHCY-0.725lnTBIL)};

Probability(CHDfemale final)= 1/{1+exp-(−15.356+0.180age-3.105HDL-C+2.938 lnHCY+0.904lnγ-GT)}.
